# Efficacy of an e-Learning Module on Endocrine Disruptors for Family Medicine Residents: Matched Before-And-After Cohort Study

**DOI:** 10.2196/89880

**Published:** 2026-05-28

**Authors:** Mathieu Diallo, Lionel Moulis, Maha Badreddine, Francois Carbonnel, Jean-Baptiste Tostain

**Affiliations:** 1 University Department of General Practice Faculty of Medicine Université de Montpellier Montpellier, Occitanie France; 2 Clinical Research and Epidemiology Unit Department of Public Health Centre Hospitalier Universitaire de Montpellier Montpellier, Occitanie France; 3 Pathogenesis and Control of Chronic and Emerging Infections INSERM, EFS, University of Antilles Université de Montpellier Montpellier, Occitanie France; 4 Department of Pedagogical Engineering and Audiovisual Production Faculty of Medicine Université de Montpellier Montpellier, Occitanie France; 5 Institut Desbrest d'Epidémiologie et de Santé Publique UMR1318 Université de Montpellier-INSERM Montpellier, Occitanie France

**Keywords:** endocrine disruptors, environmental health, primary health care, family practice, education, medical, graduate, education, distance, health behavior, internship and residency

## Abstract

**Background:**

Environmental factors account for 23% of global deaths and 25% of chronic diseases. In France, the Fourth National Environmental Health Plan prioritizes training health professionals in environmental health. Endocrine-disrupting chemicals (EDCs) are chemical substances that interfere with hormonal systems, contributing to a range of health effects. In 2024, the Primary Care and Environmental Health (PCEH) program at the University of Montpellier–Nîmes introduced an innovative e-learning module on EDCs for first-year family medicine residents.

**Objective:**

This study aimed to evaluate the impact of the PCEH e-learning module on participants’ satisfaction, knowledge, and self-reported behaviors regarding EDCs in household environments.

**Methods:**

This monocentric, matched before-and-after cohort study included all first-year family medicine residents at the University of Montpellier–Nîmes. The module, developed collaboratively by clinicians and educators, integrated interactive images, artificial intelligence–generated virtual rooms, short educational videos, games, and flash cards. Participants were assessed using pretraining and posttraining questionnaires administered immediately before and after the module. These questionnaires evaluated satisfaction (using a 5-point Likert scale), knowledge (using binary “yes” or “no” questions), and behaviors (using a 5-point Likert scale). Statistical analyses included the McNemar test for paired categorical variables and paired 2-tailed *t* tests for continuous variables, with a significance threshold set at a *P* value of less than .05.

**Results:**

This study aimed to evaluate the impact of an e-learning module on knowledge and behaviors related to endocrine disruptors. Our findings show significant improvements across all measured domains. Of 148 eligible residents, 78 (52.7%) completed both assessments over a 17-day period. Overall satisfaction was high (mean 4.0/5, SD 0.9), with positive ratings for the e-learning format (mean 4.1/5, SD 1.0) and module duration (mean 4.2/5, SD 1.0). Knowledge improved significantly, with a mean 55.56 (SD 13.54) increase in correct identification of EDCs across all substances (*P*<.001). Self-reported behaviors improved by an average of 2.13 points (95% CI 1.71-2.56) on the 5-point scale (*P*<.001), exceeding those reported in previous PCEH modules. Secondary outcomes showed high posttraining identification of at-risk populations and exposure locations, although recognition of some substances (eg, alkylphenols and phenoxyethanol) remained low.

**Conclusions:**

This innovative e-learning module significantly improved residents’ knowledge and preventive behaviors related to EDCs. These findings support the integration of environmental health training into medical curricula and highlight the potential of scalable e-learning interventions to strengthen preventive competencies in primary care.

## Introduction

Environmental health is defined as “the aspects of human health, including the quality of life, determined by the physical, chemical, biological, social, psychosocial and aesthetic factors of our environment” [1]. According to the World Health Organization, environmental factors contribute to 23% of global deaths and 25% of the global burden of chronic diseases [2,3]. In France, the Fourth National Environmental Health Plan identifies training health professionals in environmental health as one of its top priorities [4]. However, many general practitioners (GPs) report insufficient knowledge in this area and a strong need for further training [5,6].

In response to this gap, the University of Montpellier–Nîmes Faculty of Medicine launched the Primary Care and Environmental Health (PCEH) program in 2021 for family medicine residents. This blended-learning program combines e-learning modules with an annual in-person day featuring expert lectures in the morning and thematic workshops in the afternoon. Previous studies have demonstrated that e-learning can be as effective as in-person training in medical education while offering greater flexibility and accessibility [7-11].

The PCEH curriculum is updated annually with new modules. In 2024, a dedicated module on endocrine-disrupting chemicals (EDCs) was introduced for first-year family medicine residents and midwifery students. Primary care health professionals play a critical role in delivering environmental health prevention strategies, particularly during the first 1000 days of life, a period of heightened vulnerability to environmental exposures. EDCs are chemical substances or mixtures that interfere with the hormonal systems of living organisms, leading to adverse health effects in humans and wildlife [12]. Their impact is substantial: in the European Union, the estimated annual cost of EDC-related health effects is €157 billion (US $184 billion) for all inhabitants, approximately 1% of the European Union’s gross domestic product [13].

In France, the national strategy on endocrine disruptors implemented in 2021 identified EDC education for health professionals as a priority [14,15]. However, a 2012 survey of the French National Public Health Agency found that 71% of the 752 GPs questioned reported a lack of knowledge of environmental health, including endocrine disruptors [6]. Another study found that the main reason why health professionals fail to inform patients about EDCs is their own insufficient knowledge [16]. EDCs are widely present in houses [17,18], and patients increasingly seek information on this topic [19]. In the absence of formal training, health care providers often rely on mainstream media for information, which may not be evidence based [19].

Addressing this gap requires accessible, systematic education on EDCs for GP residents, enabling them to identify exposure sources and provide evidence-based preventive counseling. As with any pedagogical innovation, systematic evaluation is essential to measure its impact and guide improvement [20]. To our knowledge, no prior study has evaluated an online training module on EDCs for family medicine residents. This study aimed to assess the effectiveness of the PCEH e-learning module on EDCs in improving residents’ knowledge and self-reported preventive behaviors.

## Methods

### Study Design

This quantitative, descriptive, monocentric, matched before-and-after cohort study evaluated the knowledge and behaviors of first-year family medicine residents at the University of Montpellier–Nîmes regarding EDCs in common household settings. The study followed the STROBE (Strengthening the Reporting of Observational Studies in Epidemiology) guidelines for cohort studies (Multimedia Appendix 1).

We applied the Kirkpatrick model for training evaluation, which assesses learning outcomes across four levels: (1) *reaction*—participants’ satisfaction with the training; (2) *learning*—acquisition of knowledge, skills, and competencies; (3) *behavior*—application of learned content in a professional setting; and (4) *results*—achievement of targeted objectives, measured using predefined indicators.

Our evaluation specifically assessed participants’ ability to identify vulnerable populations, list potential health effects of EDCs, and recognize common sources of exposure. We also examined participants’ interest in environmental health, perceptions of the e-learning format, and potential thesis topics.

### Participants

The study population included all first-year family medicine residents at the University of Montpellier–Nîmes. The training was mandatory for this group. Pre- and posttraining questionnaires designed using Microsoft Forms and integrated directly into the Rise 360 platform (Articulate Global, LLC) were completed at the beginning and end of the module, respectively.

Only residents who completed both questionnaires were included in the matched analysis. After matching, data were anonymized. Although the module was also made available to midwifery students, their responses were excluded from this study.

### Intervention: Endocrine Disruptor Module

A working group was established in January 2023 comprising 1 resident, 2 university lecturers in general practice, 1 university midwife, and the audiovisual team of the University of Montpellier–Nîmes Faculty of Medicine. The module’s design was inspired by the “Endocrine disruptors – My home” website [21] (which targets the public) and was structured into 7 thematic subsections.

Pedagogical objectives were to (1) identify vulnerable populations, (2) identify primary known or potential EDCs in the home, and (3) understand the health effects of EDCs and propose preventive alternatives.

**Figure 1:**
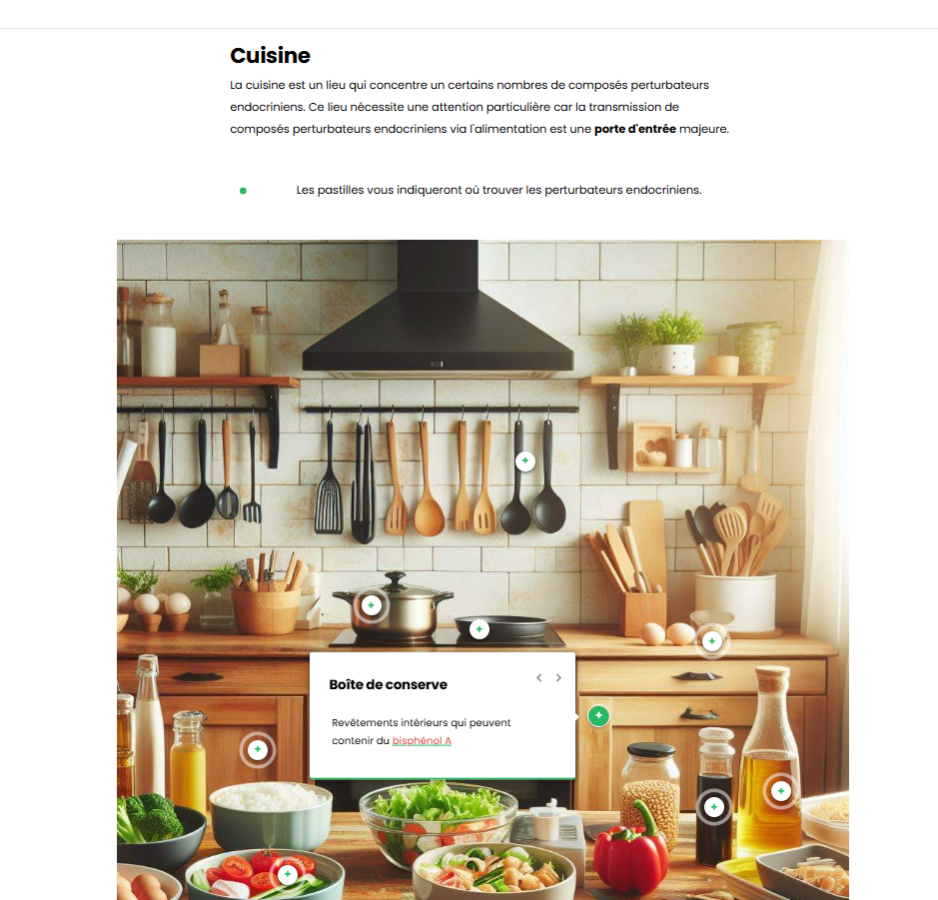
Virtual rooms within the platform.

**Figure 2:**
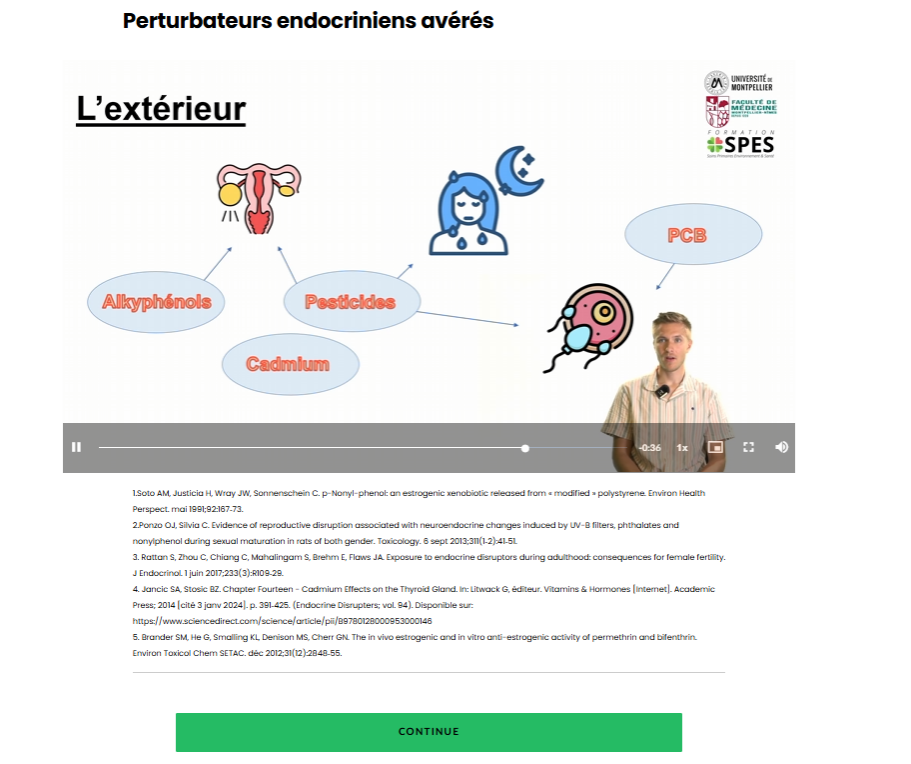
Educational videos.

**Figure 3:**
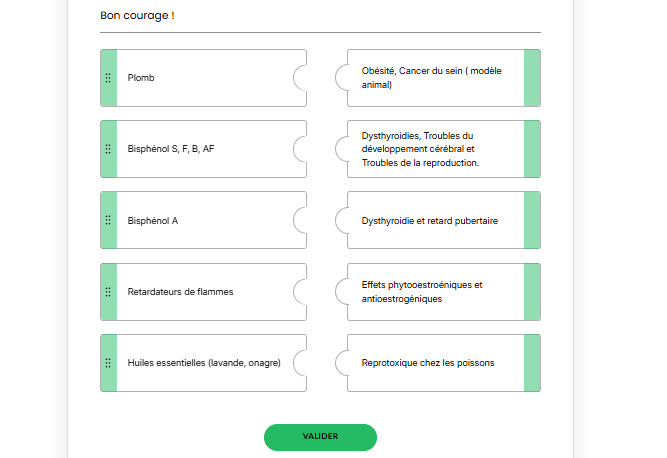
Interactive association quizzes.

Each subsection followed a consistent structure (Table 1). The module was developed using the Rise 360 platform [22], which enables the creation of interactive and adaptive e-learning content. Educational materials included (1) virtual rooms displaying household objects containing EDCs created using the Microsoft Copilot artificial intelligence tool [23] (Figure 1); (2) educational videos (≤3 minutes each) on the health effects of EDCs, produced in collaboration with the audiovisual service of the Faculty of Medicine and filmed on the set of the Department of Pedagogical Engineering and Audiovisual Production at the Faculty of Medicine (Figure 2); (3) interactive association quizzes linking EDCs to their health effects, used as tests during training (Figure 3); and (4) flash cards presenting evidence-based alternatives to reduce exposure.

**Table 1:** Structure of the e-learning module on endocrine disruptors and corresponding pedagogical methods.

Module content		Learning materials
**Introduction to EDCs^a^**		
	Pretraining questionnaire	Microsoft Forms
	Defining learning objectives	Video and text
	Definition of EDCs and their modes of action	Text and pictures
**Kitchen**		
	EDCs present	Texts and interactive visuals
	Health effects of EDCs	Video
	Quizzes	Matching exercises
	Preventive measures	Flash cards
**Bathroom**		
	EDCs present	Texts and interactive visuals
	Health effects of EDCs	Video
	Quizzes	Matching exercises
	Preventive measures	Flash cards
**Bedroom and living room**		
	EDCs present	Texts and interactive visuals
	Health effects of EDCs	Video
	Quizzes	Matching exercises
	Preventive measures	Flash cards
**Outdoors**		
	EDCs present	Texts and interactive visuals
	Health effects of EDCs	Video
	Quizzes	Matching exercises
	Preventive measures	Flash cards
**Conclusion**		
	Posttraining questionnaire	Microsoft Forms
	Summary and references	Text document

^a^EDC: endocrine-disrupting chemical.

A global summary and complete bibliography were provided at the end of the module. The training is publicly accessible [24].

### Data Collection

A literature review was conducted to identify validated pretest-posttest questionnaires for assessing knowledge and behaviors related to EDCs. No tool was fully relevant to our objectives or target population [25]. A questionnaire was then developed based on the learning objectives of the module and items from previous PCEH surveys [7,8]. It was reviewed by a panel of 3 experts, all GPs, including 2 with expertise in medical education and environmental health and 1 with specific expertise in environmental health. The experts independently assessed the items for content relevance, clarity, and alignment with the educational objectives. On the basis of their feedback, several items were revised to improve wording and reduce ambiguity. The internal consistency of the behavioral scale was assessed using the Cronbach α.

For the pretraining questionnaire, the structure was as follows: (1) sociodemographic characteristics (questions 2-7), (2) ability to identify household EDCs (yes or no; questions 8-11), (3) self-reported preventive behaviors (5-point Likert scale; questions 12, 15, 17, and 18), and (4) ancillary knowledge (multiple choice; questions 13, 14, and 16).

For the posttraining questionnaire, the knowledge and behavior items were the same (excluding sociodemographic characteristics), and the questionnaire included additional items on satisfaction with the module (questions 2-5), interest in environmental health, perception of the e-learning format, and potential thesis topics (questions 17-19).

Pseudonymization was implemented by having participants enter the first letter of their last and first names and the last 4 digits of their phone number at the start of each questionnaire, allowing for matching while preserving anonymity.

The full questionnaires are provided in Multimedia Appendices 2 and 3.

### Statistical Analysis

Data were analyzed by the study author using Microsoft Excel and the BiostatTGV statistical website [26]. Qualitative data (“yes” or “no” responses) were described as frequencies and percentages and analyzed using the McNemar chi-square test for matched samples. Regarding quantitative data (Likert scales), before-and-after differences (Δ) were calculated for each item, expressed as means, SDs, and ranges. They were analyzed using the paired Student *t* test (2-tailed). Statistical significance was set at a *P* value of less than .05. The sample size calculation was based on an assumed mean score of 2.5 out of 5 (SD 1) in the pretraining questionnaire and 3 out of 5 (SD 1) in the posttraining questionnaire, corresponding to a moderate effect size (approximate Cohen *d*=0.5). Using an α of .05 and 90% power, the required total sample size was calculated as 138 participants (69 per group) for pre- and posttest comparisons. This assumption is consistent with effect sizes commonly reported in studies evaluating educational interventions in medical education, particularly e-learning formats [9], and was considered a conservative estimate in the absence of prior data specific to endocrine disruptor training.

### Ethical Considerations

All participants provided informed consent in accordance with ethical guidelines. An impact analysis compliant with the MR-004 standard was submitted to the University of Montpellier–Nîmes’s data protection officer. Ethics committee approval was not required under the General Data Protection Regulation framework [27]. Data confidentiality was maintained per National Commission on Informatics and Liberty regulations. Anonymized data were stored on a secure, password-protected server. While participation in the training was mandatory as part of the residency program, study participation was voluntary, and residents could withdraw at any time by contacting the investigator via email. No financial compensation was provided.

## Results

### Participants

**Figure 4:**
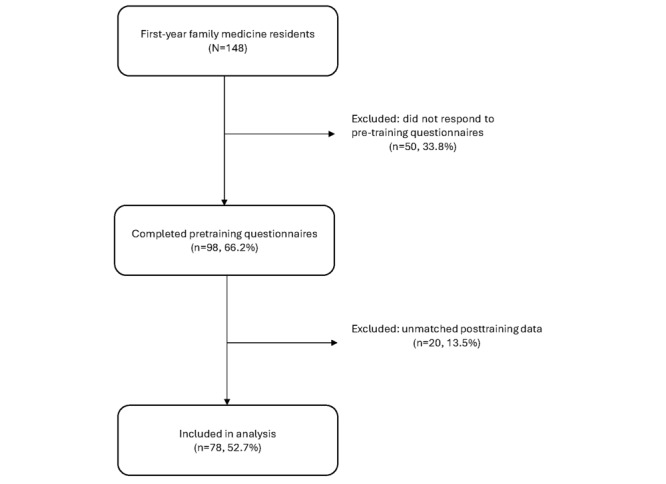
Participant flow diagram.

This study was conducted over a 17-day period (September 10-26, 2024). Of 148 eligible family medicine residents, 98 (66.2%) completed the pretraining questionnaire, and 78 (52.7%; matched participation rate) completed both the pre- and posttraining assessments (Figure 4). Sociodemographic characteristics of the study population are summarized in Table 2. The mean age was 25.82 (SD 1.5) years, and 73.1% (57/78) of the participants were women. Only 11.5% (9/78) reported prior training in environmental health.

**Table 2:** Sociodemographic characteristics of the participants (N=78).

Characteristic		Values
**Sex, n (%)**		
	Female	57 (73.1)
	Male	21 (26.9)
Age (y), mean (SD)		25.82 (1.5)
Had children, n (%)		2 (2.6)
Previous training in environmental health, n (%)		9 (11.5)
**Main sources of information on environmental health, n (%)**		
	Media	48 (61.5)
	Websites and forums	13 (16.7)
	Encyclopedic sites	14 (17.9)
	Official websites	17 (21.8)
	Relatives	22 (28.2)
	Health professionals	25 (32.1)
	Patient, consumer, or environmental protection associations	5 (6.4)
	Learned at the University of Montpellier–Nîmes	12 (15.4)
	Did not stay up-to-date	23 (29.5)
	Not interested	4 (5.1)

### Satisfaction

Overall satisfaction with the 4 modules (kitchen, bathroom, bedroom and living room, and outdoors) was rated at a mean of 4.0/5 (SD 0.9). The suitability of the e-learning format was rated at a mean of 4.1/5 (SD 1.0), and the extent to which the training achieved its objectives was rated at a mean of 4.3/5 (SD 0.8). Regarding module length, 77% (60/78) of the participants were satisfied, including 47% (37/78) who were completely satisfied and 30% (23/78) who were moderately satisfied; 15% (12/78) were indifferent; and 7% (6/78) were little or not satisfied. Detailed satisfaction data are provided in Multimedia Appendix 6.

### Knowledge Outcomes

**Figure 5:**
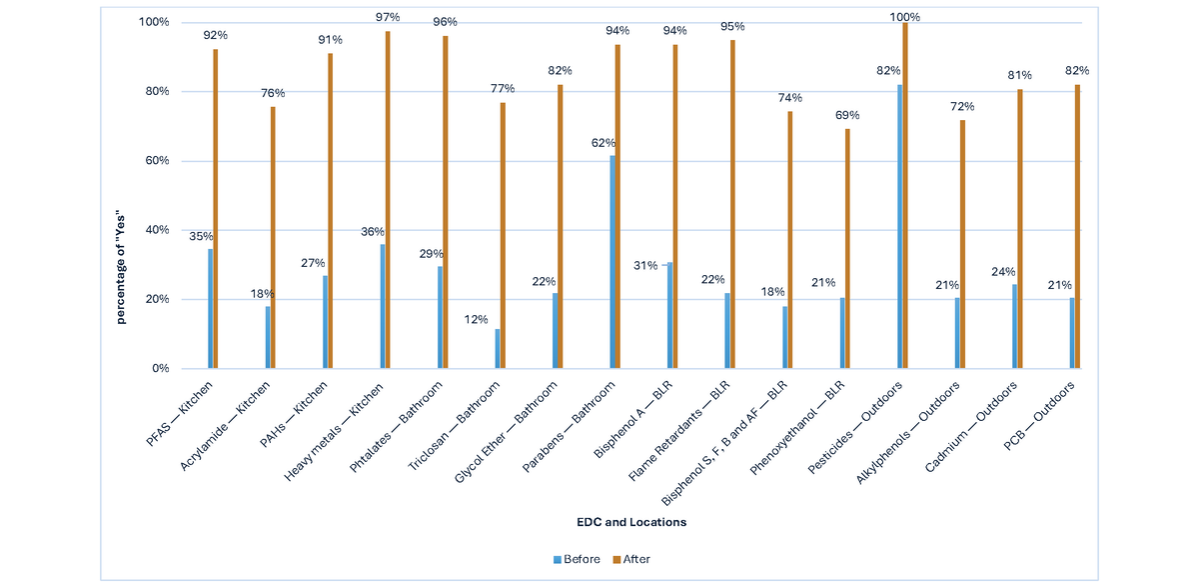
Percentage of correct identification of household endocrine-disrupting chemicals (EDCs) before and after the training. Responses to binary (“yes” or “no”) questions. All differences were statistically significant at *P*<.001 (McNemar test). BLR: bedroom and living room; PAH: polycyclic aromatic hydrocarbon; PCB: polychlorinated biphenyl; PFAS: per- and polyfluoroalkyl substance.

Identification rates for all 16 EDCs improved significantly (*P*<.001; Figure 5). Before the training, correct identification ranged from 12% (9/78) to 36% (28/78) for 14 of the 16 substances. After the training, rates increased to 69% (54/78) to 97% (76/78). The largest absolute increases were observed for pesticides (64/78, 82.1% before vs 78/78, 100% after) and parabens (30/78, 38.5% before vs 73/78, 93.6% after). The mean overall improvement in correct identification across all EDCs was 55.56 (SD 13.54) percentage points. Detailed data are available in Multimedia Appendix 5.

### Behavioral Outcomes

**Figure 6:**
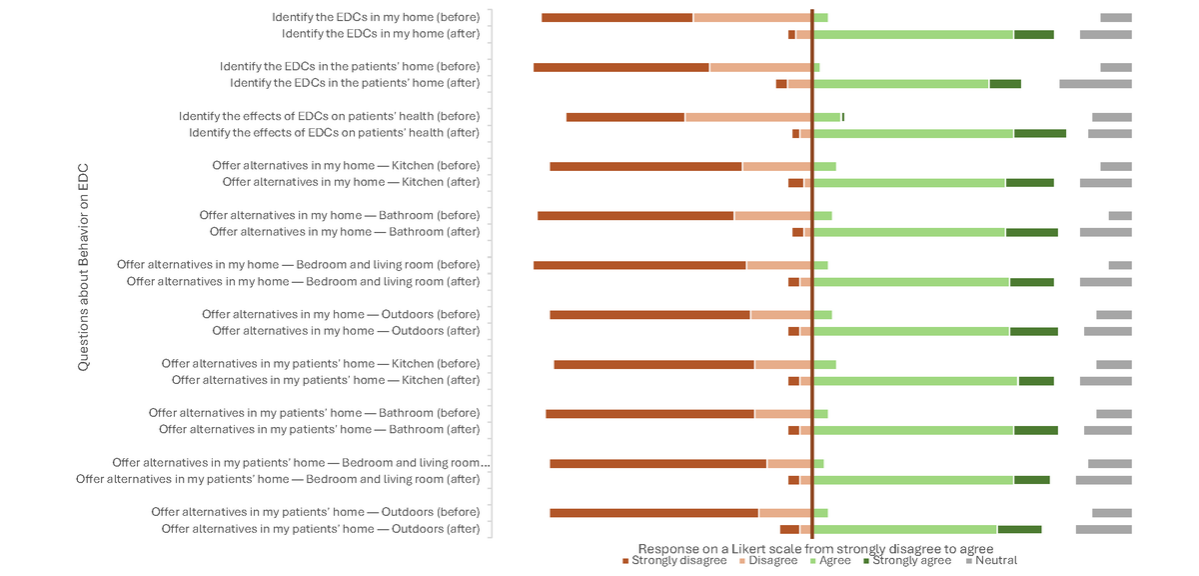
Pretraining and posttraining self-reported behaviors related to endocrine disruptors, measured on a 5-point Likert scale (1=“strongly disagree”; 5=“strongly agree”). All differences were statistically significant at *P*<.001 (Student t test). EDC: endocrine-disrupting chemical.

The mean pretraining behavior score on the 5-point Likert scale was 1.63 (SD 0.89). After the training, the mean score increased to 3.77 (SD 0.88), representing a significant mean improvement of 2.13 points (95% CI 1.71-2.56; *P*<.001; Figure 6). This pattern was consistent across all household settings (kitchen, bathroom, bedroom and living room, and outdoors) and for both self-assessment items (“in my home” and “in patients’ homes”).

The internal consistency of the behavioral scale was assessed using the Cronbach α, which was 0.91, indicating excellent internal consistency.

Detailed data are available in Multimedia Appendix 4.

### Secondary Criteria

#### Identification of At-Risk Populations

Knowledge of at-risk populations was high before the training (72/78, 92%) and remained high after the training (76/78, 97%; Table 3).

**Table 3:** Additional knowledge on endocrine disruptors: at-risk populations, associated health effects, and household locations (N=78).

		Before the training, n (%)	After the training, n (%)
**At-risk populations**			
	Embryonic life and pregnancy	78 (100)	78 (100)
	Early childhood	69 (88.5)	74 (94.9)
	Adolescence and puberty	68 (87.2)	74 (94.9)
**Health effects**			
	Obesity	58 (74.4)	77 (98.7)
	Diabetes	57 (73.1)	78 (100)
	Fertility disorders	77 (98.7)	77 (98.7)
	Behavioral disorders	49 (62.8)	63 (80.8)
	Thyroid disorders	74 (94.9)	77 (98.7)
	Congenital malformations	72 (92.3)	73 (93.6)
	Cancer	69 (88.5)	71 (91)
**Location of EDCs^a^**			
	Meat	60 (76.9)	78 (100)
	Fish	62 (79.5)	78 (100)
	Vegetables	57 (73.1)	76 (97.4)
	Processed products	75 (96.2)	77 (98.7)
	Cosmetics	76 (97.4)	77 (98.7)
	Bedding	51 (65.4)	75 (96.2)
	Clothes	60 (76.9)	76 (97.4)
	Kitchen utensils	60 (76.9)	77 (98.7)
	Paint	76 (97.4)	77 (98.7)
	Diapers	67 (85.9)	75 (96.2)
	Sanitary towels	73 (93.6)	77 (98.7)
	Fertilizer	69 (88.5)	73 (93.6)

^a^EDC: endocrine-disrupting chemical.

#### Identification of Associated Pathologies

Before the training, obesity, diabetes, and behavioral disorders were the least frequently recognized EDC-associated conditions. After the training, all items increased in recognition, although the absolute gain for behavioral disorders was smaller than for other conditions (Table 3).

#### Localization of EDCs

After the training, the ability to identify the location of EDCs reached 98% (77/78) for most listed items, with improvements across all categories (Table 3).

#### Interest in Environmental Health and Thesis Topics

After the training, 82.1% (64/78) of the participants expressed interest in further environmental health training during their clerkship, and 78.2% (61/78) expressed interest in further training during residency. The preferred format for future training was in-person sessions (43/78, 55%). Despite the increased interest, 85.9% (67/78) reported no intention to conduct a thesis on environmental health, and 94.9% (74/78) reported no interest in a thesis specifically on EDCs. The option of developing a thesis in an e-learning format was rejected by 93.6% (73/78) of respondents.

## Discussion

### Main Results

Participants reported high overall satisfaction (mean 4.0/5, SD 0.9) across all module sections, with positive ratings for the e-learning format (mean 4.1/5, SD 1.0) and module duration (mean 4.2/5, SD 1.0). Compared with earlier PCEH modules, this version integrated short videos, interactive pictures, and flash cards—pedagogical tools known to enhance learner engagement and satisfaction [28]. Previous literature suggests that high satisfaction correlates with greater learning gains [29].

Knowledge improved markedly, with a mean 56% increase in correct identification of EDCs across all substances (*P*<.001), exceeding gains reported in prior PCEH evaluations [7,8] and aligning with results from other environmental health training programs [30]. By the end of the module, participants could identify numerous EDCs in common household environments, enabling them to detect these exposures during consultations or home visits.

Self-reported behaviors also improved significantly, with a mean increase of 2.13 points (95% CI 1.71-2.56) on a 5-point Likert scale—greater than improvements observed in previous PCEH studies (0.74 and 1.1 points) [7,8]. These gains fulfill the third pedagogical objective, equipping participants to propose evidence-based alternatives to reduce exposure. Such counseling can contribute to exposure-based prevention strategies and may help mitigate cumulative risks from multiple substances (“cocktail effect”) [31] while balancing reduction efforts with avoidance of undue patient anxiety [32].

Baseline results confirmed limited access to reliable information on EDCs, with the media as the primary source [7,16]. The asynchronous e-learning format allowed for broad participation across the Occitania region (41/78, 52% of the cohort), with sociodemographic characteristics comparable to those in previous PCEH studies [7,8].

Gains in identification were smaller for pesticides and parabens, likely reflecting high baseline recognition (64/78, 82% and 48/78, 62%, respectively), suggesting a ceiling effect. However, some heterogeneity in learning outcomes was observed. Some items showed lower recognition rates even after the intervention, particularly behavioral disorders, alkylphenols, and phenoxyethanol. Several explanations may account for this pattern. First, these topics may be less familiar to general practice residents, either because they are less frequently encountered in routine clinical practice or because they are less emphasized during initial medical training. Second, certain substances, such as alkylphenols and phenoxyethanol, involve more technical knowledge and may be more difficult to retain following a single e-learning session. Third, health effects such as behavioral disorders may be perceived as multifactorial and less directly attributable to endocrine disruptors, which could limit participants’ confidence in identifying these associations. These findings highlight potential areas for improvement in future versions of the module, suggesting the need to reinforce these specific topics through more explicit teaching, clinical case illustrations, or repeated exposure**.** This pattern also suggests that a single exposure to the content may not be sufficient for more complex or less salient topics.

Secondary outcomes showed strong identification of critical exposure periods and locations, confirming the first pedagogical objective and reinforcing prior training. The weaker association between EDCs and the “behavioral disorder” item highlights a target for future module updates.

### Strengths and Limitations

A major strength of this study is the use of a structured pedagogical framework (Kirkpatrick model) combined with an innovative instructional design integrating interactive media, artificial intelligence–generated visuals, and professionally produced short videos. The module was developed collaboratively by educators, clinicians, and audiovisual specialists, ensuring both scientific accuracy and pedagogical quality.

The evaluation benefited from a matched before-and-after design and covered all participants in a defined academic cohort. The sociodemographic profile was consistent with those of previous cohorts, supporting generalizability to similar populations.

However, the study has limitations. The 17-day data collection window limited recruitment, and results are based on 52.7% (78/148) of eligible residents. The monocentric setting limits external validity, and midwifery students were excluded due to insufficient responses. Parenthood, a potential confounder [33], was rare (2/78, 2.6%) and not analyzed.

Outcomes were assessed immediately after the training, introducing possible recall bias and precluding assessment of long-term retention; self-reporting also raises the risk of social desirability bias. Objective measures such as clinical case assessments or objective structured clinical examinations could strengthen future evaluations.

Although the internal consistency was excellent, further psychometric validation (eg, factor analysis) would be needed to fully validate the scale.

A key limitation relates to the higher levels of the Kirkpatrick model, particularly the assessment of behavior changes and impact on clinical practice. As participants were early-stage residents, their opportunities to apply newly acquired knowledge in real clinical settings may have been limited. While early exposure to environmental health concepts may increase awareness, it may not immediately translate into observable changes in practice.

However, this also highlights opportunities for reinforcing learning throughout residency. Future educational strategies could include integrating environmental health considerations into clinical reasoning, for example, by systematically exploring occupational and environmental exposures during patient history taking or by documenting potential environmental determinants of health in medical records. More broadly, longitudinal approaches combining repeated exposure, clinical case discussions, and practical application may help facilitate the translation of knowledge into practice. This aligns with educational models emphasizing spiral curricula and progressive integration of competencies over time [34].

### Perspectives

The strong results support the continued integration of this e-learning module into the PCEH curriculum and its potential extension to other health professionals involved in environmental health prevention. Scaling could involve multidisciplinary health centers, territorial health professional communities, and continuing education programs, with evaluation of reproducibility across professional groups.

Incorporating a standardized EDC assessment tool into the module could help professionals prioritize intervention areas during consultations. Additional content could address substances with persistently low recognition rates (eg, alkylphenols and phenoxyethanol) through simplified explanations or interactive case scenarios.

Given participants’ preference for some in-person learning, hybrid formats could be explored—for example, pairing e-learning with expert-led workshops during annual PCEH conferences. Additional modules, such as those on EDCs in pharmaceuticals, are planned for 2025.

Although interest in conducting theses on EDCs or environmental health remained low, earlier exposure in the medical curriculum, thesis mentorship, and targeted workshops could increase engagement. As first-year residents must now select thesis topics by the end of year 1, introducing this training earlier—during the second cycle—could enhance its influence on research topic selection. Tracking the number of theses on environmental health since the PCEH’s inception could help assess its research impact.

Finally, integration with prevention initiatives (eg, Desbrest Institute of Epidemiology and Public Health consultations, ASALEE (Action de Santé Libérale En Équipe) nurse protocols, or standardized prevention assessments [35]) and with the Platform for Data in Primary Care [36] could strengthen monitoring and targeted interventions, particularly for individuals of childbearing age.

## Data Availability

The datasets generated or analyzed during this study are available from the corresponding author on reasonable request.

## Appendix

Multimedia Appendix 1 STROBE checklist for reporting cohort studies.

Multimedia Appendix 2 Pretraining questionnaire.

Multimedia Appendix 3 Posttraining questionnaire.

Multimedia Appendix 4 Participant satisfaction.

Multimedia Appendix 5 Number of positive responses for identification of household endocrine disruptors before and after the training (N=78).

Multimedia Appendix 6 Mean pretraining and posttraining scores for Likert-scale behavior questions and corresponding mean differences.

## Abbreviations

EDC: endocrine-disrupting chemical
GP: general practitioner
PCEH: Primary Care and Environmental Health
STROBE: Strengthening the Reporting of Observational Studies in Epidemiology
