# Supplementary material for: Efficacy of an e-Learning Module on Endocrine Disruptors for Family Medicine Residents: Matched Before-And-After Cohort Study
Source: JMIR Form Res. 2026 May 28;10:e89880. doi: 10.2196/89880 (PMC13261164; doi:10.2196/89880)
Supplement: Multimedia Appendix 3 [file formative_v10i1e89880_app3.docx]

**Post-Training Questionnaire on Endocrine Disruptors**

**Introduction**

As part of the SPES Module, we have created an E-learning course on endocrine disruptors. We aim to evaluate this training by measuring its impact on the knowledge and behaviors of students. This study is part of Mathieu DIALLO's thesis. The following questionnaire aims to collect demographic data and your knowledge. **Your identifying data will remain anonymous and destroyed.**

The information collected in this study is recorded in a computerized file by Mathieu DIALLO to evaluate the performance of the training, its impact on the knowledge and behaviors of the interviewed individuals, and to improve the E-learning tool. The collected data will be communicated only to the following recipients: Mathieu DIALLO, Jean-Baptiste Tostain. The data will be kept until the thesis is defended. This processing is necessary for the execution of a public service mission, to understand the work in the context of a training project.

Refer to the CNIL Website for more information about your rights. In accordance with the General Data Protection Regulation (EU Regulation 2016-679 of April 27, 2016) and the French Data Protection Act (Law No. 78-17 of January 6, 1978, as amended), you have:

- A right of access, rectification, and erasure of your personal data;
- A right to withdraw your consent at any time;
- A right to limit and oppose the processing of your personal data.

To exercise these rights or to report any anomaly, you can contact the investigator of this study at: [mathieu.diallo@etu.umontpellier.fr](mailto:mathieu.diallo@etu.umontpellier.fr). If necessary, you can also file a complaint with the National Commission for Informatics and Liberties (CNIL).

By responding to this questionnaire, you consent to the processing of this data.

For any questions, complaints, or to withdraw from this study at any time, you can contact Mathieu DIALLO by email: [mathieu.diallo@etu.umontpellier.fr](mailto:mathieu.diallo@etu.umontpellier.fr).

Estimated time to complete the questionnaire: **10 minutes**.

**1. Please indicate the initials of your last name, first name, and the last 4 digits of your phone number.**

**2. Indicate your level of satisfaction with the online training on endocrine disruptors for each module.**

| **Module** | **1 - Not at all satisfied** | **2 - Dissatisfied** | **3 - Indifferent** | **4 - Satisfied** | **5 - Very satisfied** |
| --- | --- | --- | --- | --- | --- |
| The kitchen | ☐ | ☐ | ☐ | ☐ | ☐ |
| The bathroom | ☐ | ☐ | ☐ | ☐ | ☐ |
| The bedroom and living room | ☐ | ☐ | ☐ | ☐ | ☐ |
| Outdoors | ☐ | ☐ | ☐ | ☐ | ☐ |

**3. The E-Learning format was suitable for training on endocrine disruptors.**

| **1 - Not at all in agreement** | **2 - Not in agreement** | **3 - Indifferent** | **4 - In agreement** | **5 - Completely in agreement** |
| --- | --- | --- | --- | --- |
| ☐ | ☐ | ☐ | ☐ | ☐ |

**4. The training met the pedagogical objectives presented at the beginning of the training.**

| **1 - Not at all in agreement** | **2 - Not in agreement** | **3 - Indifferent** | **4 - In agreement** | **5 - Completely in agreement** |
| --- | --- | --- | --- | --- |
| ☐ | ☐ | ☐ | ☐ | ☐ |

**5. The duration of the modules and videos was appropriate.**

| **1 - Not at all in agreement** | **2 - Not in agreement** | **3 - Indifferent** | **4 - In agreement** | **5 - Completely in agreement** |
| --- | --- | --- | --- | --- |
| ☐ | ☐ | ☐ | ☐ | ☐ |

**6. Can you identify the proven or potential endocrine disruptors that can be found in the kitchen?**

| **Endocrine Disruptor** | **Yes** | **No** |
| --- | --- | --- |
| Perfluorinated or PFAS compounds | ☐ | ☐ |
| Acrylamide | ☐ | ☐ |
| Polycyclic aromatic hydrocarbons (PAH) | ☐ | ☐ |
| Heavy metals | ☐ | ☐ |

**7. Can you identify the proven or potential endocrine disruptors that can be found in the bathroom?**

| **Endocrine Disruptor** | **Yes** | **No** |
| --- | --- | --- |
| Phthalates | ☐ | ☐ |
| Triclosan | ☐ | ☐ |
| Ethylene glycol | ☐ | ☐ |
| Parabens | ☐ | ☐ |

**8. Can you identify the proven or potential endocrine disruptors that can be found in the bedroom and living room?**

| **Endocrine Disruptor** | **Yes** | **No** |
| --- | --- | --- |
| Bisphenol A | ☐ | ☐ |
| Flame retardants | ☐ | ☐ |
| Bisphenol S, F, B, AF | ☐ | ☐ |
| Phenoxyethanol | ☐ | ☐ |

**9. Can you identify the proven or potential endocrine disruptors that can be found outdoors?**

| **Endocrine Disruptor** | **Yes** | **No** |
| --- | --- | --- |
| Pesticides | ☐ | ☐ |
| Alkylphenols | ☐ | ☐ |
| Cadmium | ☐ | ☐ |
| Polychlorinated biphenyls or PCB | ☐ | ☐ |

**10. Do you know how to identify the effects of endocrine disruptors on human health?**

| **1 - Not at all in agreement** | **2 - Not in agreement** | **3 - Indifferent** | **4 - In agreement** | **5 - Completely in agreement** |
| --- | --- | --- | --- | --- |
| ☐ | ☐ | ☐ | ☐ | ☐ |

**11. To which pathologies can endocrine disruptors be linked?**

- ☐ Obesity
- ☐ Diabetes
- ☐ Fertility issues
- ☐ Behavioral disorders
- ☐ Thyroid disorders
- ☐ Congenital malformations
- ☐ Cancer

**12. In your opinion, which are the 3 most vulnerable populations to endocrine disruptors?**

- ☐ Embryonic life / pregnancy
- ☐ Early childhood
- ☐ Adolescence / puberty
- ☐ Adults
- ☐ Elderly people

**13. I feel capable of detecting endocrine disruptors in objects present in different rooms of the house:**

| **Location** | **1 - Not at all in agreement** | **2 - Not in agreement** | **3 - Indifferent** | **4 - In agreement** | **5 - Completely in agreement** |
| --- | --- | --- | --- | --- | --- |
| My home | ☐ | ☐ | ☐ | ☐ | ☐ |
| Patients' homes | ☐ | ☐ | ☐ | ☐ | ☐ |

**14. In which products do you think you can find endocrine disruptors?**

- ☐ Meats
- ☐ Fish
- ☐ Vegetables
- ☐ Processed products
- ☐ Cosmetics
- ☐ Literature
- ☐ Clothes
- ☐ Kitchen utensils
- ☐ Paint
- ☐ Mattresses
- ☐ Hygiene products

**15. Following this training, I feel capable of offering alternative solutions and giving advice to limit endocrine disruptors in my habitat:**

| **Location** | **1 - Not at all in agreement** | **2 - Not in agreement** | **3 - Indifferent** | **4 - In agreement** | **5 - Completely in agreement** |
| --- | --- | --- | --- | --- | --- |
| In the kitchen | ☐ | ☐ | ☐ | ☐ | ☐ |
| In the bathroom | ☐ | ☐ | ☐ | ☐ | ☐ |
| In the bedroom and living room | ☐ | ☐ | ☐ | ☐ | ☐ |
| Outdoors | ☐ | ☐ | ☐ | ☐ | ☐ |

**16. Following this training, I feel capable of offering alternative solutions and giving advice to limit endocrine disruptors in the environment of my patients:**

| **Location** | **1 - Not at all in agreement** | **2 - Not in agreement** | **3 - Indifferent** | **4 - In agreement** | **5 - Completely in agreement** |
| --- | --- | --- | --- | --- | --- |
| In the kitchen | ☐ | ☐ | ☐ | ☐ | ☐ |
| In the bathroom | ☐ | ☐ | ☐ | ☐ | ☐ |
| In the bedroom and living room | ☐ | ☐ | ☐ | ☐ | ☐ |
| Outdoors | ☐ | ☐ | ☐ | ☐ | ☐ |

**17. More training in environmental health would be beneficial.**

| **Yes** | **No** |
| --- | --- |
| ☐ | ☐ |

- ☐ Online
- ☐ In-person

**18. Would you like a face-to-face format to complete your training on endocrine disruptors?**

| **Yes** | **No** |
| --- | --- |
| ☐ | ☐ |

**19. Are you considering doing a thesis on:**

| **Yes** | **No** |
| --- | --- |
| Endocrine disruptors ☐ | ☐ |
| Environmental health ☐ | ☐ |
| Creating an E-learning with or without podcast/video ☐ | ☐ |
