# Supplementary material for: Efficacy of an e-Learning Module on Endocrine Disruptors for Family Medicine Residents: Matched Before-And-After Cohort Study
Source: JMIR Form Res. 2026 May 28;10:e89880. doi: 10.2196/89880 (PMC13261164; doi:10.2196/89880)
Supplement: Multimedia Appendix 4 [file formative_v10i1e89880_app4.docx]

| **Item** | **Mean (SD)** |
| --- | --- |
| **Satisfaction with module parts** |  |
| Kitchen | 4.1 (0.8) |
| Bathroom | 4.0 (0.9) |
| Bedroom and living room | 4.0 (0.9) |
| Outdoors | 4.0 (0.9) |
| **Overall satisfaction** |  |
| Format of the e-learning module | 4.1 (1.0) |
| Achievement of expected objectives | 4.3 (0.8) |
| Appropriateness of module duration | 4.2 (1.0) |
