# Supplementary material for: Efficacy of an e-Learning Module on Endocrine Disruptors for Family Medicine Residents: Matched Before-And-After Cohort Study
Source: JMIR Form Res. 2026 May 28;10:e89880. doi: 10.2196/89880 (PMC13261164; doi:10.2196/89880)
Supplement: Multimedia Appendix 5 [file formative_v10i1e89880_app5.docx]

|  | Before  n (%) | After  n (%) | Difference (%) |
| --- | --- | --- | --- |
| Kitchen |  |  |  |
| PFAS | 27 (34.6) | 72 (92.3) | +57.7 |
| Acrylamide | 14 (17.9) | 59 (75.6) | +57.7 |
| PAHs | 21 (26.9) | 71 (91.0) | +64.1 |
| Heavy metals | 28 (35.9) | 76 (97.4) | +61.5 |
| Bathroom |  |  |  |
| Phthalates | 23 (29.5) | 75 (96.2) | +66.7 |
| Triclosan | 9 (11.5) | 60 (76.9) | +65.4 |
| Glycol ethers | 17 (21.8) | 64 (82.1) | +60.3 |
| Parabens | 48 (61.5) | 73 (93.6) | +55.1 |
| Bedroom and living room |  |  |  |
| Bisphenol A | 24 (30.8) | 73 (93.6) | +62.8 |
| Flame retardants | 17 (21.8) | 74 (94.9) | +73.1 |
| Bisphenol S, B, F, AF | 14 (17.9) | 58 (74.4) | +56.5 |
| Phenoxyethanol | 16 (20.5) | 54 (69.3) | +48.7 |
| Outdoors |  |  |  |
| Pesticides | 64 (82.1) | 78 (100) | +17.9 |
| Alkylphenols | 16 (20.5) | 56 (71.8) | +51.3 |
| Cadmium | 19 (24.4) | 63 (80.8) | +56.4 |
| PCBs | 16 (20.5) | 64 (82.1) | +61.6 |

All differences were statistically significant at P<.001 (McNemar test).
