# Supplementary material for: Efficacy of an e-Learning Module on Endocrine Disruptors for Family Medicine Residents: Matched Before-And-After Cohort Study
Source: JMIR Form Res. 2026 May 28;10:e89880. doi: 10.2196/89880 (PMC13261164; doi:10.2196/89880)
Supplement: Multimedia Appendix 6 [file formative_v10i1e89880_app6.docx]

| **Item** | **Pre-training**  **mean (SD)** | **Post-training**  **mean (SD)** | **Mean difference [95% CI]** |
| --- | --- | --- | --- |
| Identify EDCs in my home | 1.73 (0.85) | 3.78 (0.83) | 2.05 [1.85; 2.25] |
| Identify EDCs in patients' Home | 1.60 (0.78) | 3.60 (0.92) | 2.00 [1.79; 2.21] |
| Identify the health effects of EDCs | 1.97 (0.99) | 3.87 (0.83) | 1.90 [1.62; 2.17] |
| Suggest alternatives in my home: kitchen | 1.58 (0.89) | 3.83 (0.88) | 2.24 [2.00; 2.48] |
| Suggest alternatives in my home: bathroom | 1.65 (0.96) | 3.78 (0.93) | 2.13 [1.88; 2.37] |
| Suggest alternatives in my home: bedroom and living room | 1.51 (0.85) | 3.78 (0.88) | 2.27 [2.04; 2.50] |
| Suggest alternatives in my home: outdoors | 1.62 (0.93) | 3.81 (0.88) | 2.19 [1.95; 2.43] |
| Suggest alternatives in patients' homes: kitchen | 1.56 (089) | 3.79 (0.87) | 2.12 [1.88; 2.35] |
| Suggest alternatives in patients' homes: bathroom | 1.64 (0.97) | 3.76 (0.86) | 2.23 [1.99; 2.47] |
| Suggest alternatives in patients' homes: bedroom and living room | 1.54 (0.88) | 3.74 (0.86) | 2.21 [1.97; 2.44] |
| Suggest alternatives in patients' homes: outdoors | 1.58 (0.9) | 3.69 (0.98) | 2.12 [1.87; 2.36] |

All differences were statistically significant at P<.001 ( Student's test).
